# Bacterial aetiological agents of intra-amniotic infections and preterm birth in pregnant women

**DOI:** 10.3389/fcimb.2013.00058

**Published:** 2013-10-16

**Authors:** George L. Mendz, Nadeem O. Kaakoush, Julie A. Quinlivan

**Affiliations:** ^1^School of Medicine, Sydney, The University of Notre Dame AustraliaDarlinghurst, NSW, Australia; ^2^School of Biotechnology and Biomolecular Sciences, The University of New South WalesKensington, NSW, Australia; ^3^School of Medicine, Fremantle, The University of Notre Dame AustraliaFremantle, WA, Australia

**Keywords:** microbiome, intra-uterine infection, preterm birth

## Abstract

Infection-related preterm birth is a leading cause of infant mortality and morbidity; knowledge of bacterial populations invading the amniotic cavity and the routes of invasion is required to make progress in the prevention of preterm birth. Significant advances have been made in understanding bacterial communities in the vagina, but much less studied are intra-uterine bacterial populations during pregnancy. A systematic review of data published on the intra-uterine microbiome was performed; molecular information and summaries of species found in healthy individuals and in women with diagnosed infections served to construct a database and to analyse results to date. Thirteen studies fulfilled the review's inclusion criteria. The data of various investigations were collated, organized, and re-analyzed to achieve a more comprehensive understanding of microbial populations in the intra-amniotic space. The most common intra-amniotic bacterial taxa were species that can colonies the vagina in health and disease; there were others associated with the habitats of the mouth, gastrointestinal tract, and respiratory tract. The results suggest a central role for the ascending route of infections during pregnancy, and point to a possible secondary contribution via haematogenous invasion of the intra-amniotic space. The complete census of the intra-uterine microbiome awaits completion.

## Introduction

“Infections of mothers and their babies (both *in utero* and *ex utero*) are a major global challenge” (Hussein et al., 2011). Preterm birth (PTB) is the second largest direct cause of deaths in children younger than 5 years (Blencowe et al., 2012); it is a major cause of perinatal mortality and serious neonatal morbidity, and moderate to severe childhood disability in developed and developing countries (Lawn et al., 2005; Hemminki et al., 2007; Jacobsson, 2007). The burden of PTB is substantial and increased between 1990 and 2010 in developing and developed countries with reliable data (Blencowe et al., 2012). Length of gestation is considered to be a key indicator of infant health, and PTB is associated with poorer health outcomes in babies.

Premature deliveries can be classified into two broad groups: spontaneous and iatrogenic. The majority of PTB occur spontaneously as a result of preterm labor or preterm premature rupture of membranes. Spontaneous preterm delivery occurs in ~12% of births in developed countries (Pretorius et al., 2007) and 14% worldwide (Pararas et al., 2006). Iatrogenic PTB may be secondary to other complications of pregnancy such as preeclampsia, intrauterine growth restriction, abruptio placenta, or placenta praevia (Muglia and Katz, 2010).

“For much of the 20th century, PTB, defined as birth at less than 37 completed weeks of gestation, was viewed as an unpredictable and inevitable fact of life. Medical efforts thus focused on ameliorating the consequences of prematurity rather than preventing its occurrence. This approach resulted in improved neonatal outcomes, but it remains costly in terms of both the suffering of infants and their families and the economic burden on society.” (Muglia and Katz, 2010). The burden of PTB increased during the last 30 years owing to significant improvements in neonatal care that made possible the survival of very preterm infants and resulted in a lowering of the threshold for preterm Caesarean delivery. Other factors that have contributed to higher rates of PTB are the multiple gestations arising from the use of assisted reproductive technologies, advanced maternal age, and improvements in obstetrics outcomes of surgical interventions to manage invasive lesions (Muglia and Katz, 2010).

**Figure 1:**
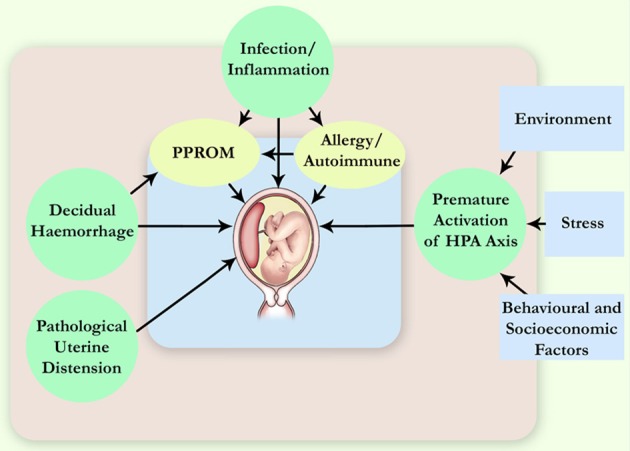
**Pathophysiological mechanisms leading to spontaneous PTB.** More than one pathway can lead to the same immediate cause of premature delivery, for example, the preterm pre-labor rupture of membranes (PPROM). HPA axis, hypothalamic-pituitary-adrenal axis.

The aetiology of PTB is multifactorial, and various factors have been identified as contributors to spontaneous PTB (Gracie et al., 2011), e.g., genetic, infection and inflammation, decidual haemorrhage, and environmental, behavioral and social stress (Figure 1). Infections have been long suspected to be the underlying cause of idiopathic PTB, and microbial intra-uterine infection is a confirmed leading cause of PTB. In particular, bacterial invasion of the amniotic cavity (BIAC) is the chief cause of neonatal mortality worldwide (Gonçalves et al., 2002; Lawn et al., 2005). Currently, there is overwhelming evidence to implicate infection in up to 40% of PTB cases, including intra-uterine (Ganu et al., 2013) and vaginal (Hyman et al., 2013) infections. Intra-amniotic infections are present in ~50% of all pregnancies that result in PTB, and the earlier the gestational age at delivery, the higher the frequency of intra-amniotic infection (Burd et al., 2012).

A study early in the twentieth Century supported the view that under normal circumstances and prior to labor, the amniotic cavity was sterile (Harris and Brown, 1927). The gold standard for identification of intrauterine infection has been the isolation of microbes in amniotic fluid sampled by amniocentesis. Owing to the finding that the amniotic fluid of less than 1% of women in labor at term contains culturable bacteria (Romero et al., 2002), historically the isolation of any microbes from the amniotic fluid was considered a pathological finding. However, investigations of the intra-uterine flora of women giving birth prematurely based on non-cultivation taxon-specific PCR analyses, as well as more recent studies employing non-cultivation broad-range PCR methods have demonstrated the presence of microflora in the intra-amniotic cavity even in the absence of any signs of infection (DiGiulio, 2012). The results of standard microbiological studies suggest that intrauterine infection accounts for as much as 25–45% of spontaneous PTB (Zhou et al., 2010); but employing molecular techniques, bacterial footprints have been detected in as many as 60% of women delivering preterm (Gardella et al., 2004).

The sequence of events leading to PTB, progressing from intrauterine infection to pro-inflammatory cytokine activation, prostaglandin production, premature contractions, cervical changes, and premature delivery has been comprehensively studied on non-human primates (Adams Waldorf et al., 2011). In humans, the colonization of microbes and/or inflammation of the chorio-decidual interface can induce the production of a cascade of cytokines that result in an inflammatory response (Muglia and Katz, 2010). Bacteria also can have a more direct role in the pathogenesis of PTB by producing enzymes that degrade fetal membranes, or by inducing the synthesis and release of uterotonins such as prostaglandins, able to stimulate uterine contractions and cause preterm labor (Keelan et al., 2003; Lockwood, 2013).

Notwithstanding the evidence, current knowledge of BIAC is insufficient to develop effective strategies to prevent infection-related PTB because the prevalence, methods of diagnosis, pathogenicity mechanisms, and host susceptibilities to various bacteria require further investigations (DiGiulio, 2012). A necessary step to address these knowledge gaps is to obtain a complete understanding of the diverse microbial taxa involved in BIAC.

Pathogens may gain access to the amniotic cavity and fetus by ascending migration of the vaginal flora, haematogenous dissemination through the placenta, retrograde seeding from the peritoneal cavity through the Fallopian tubes, or iatrogenic introduction at the time of invasive procedures (Goldenberg et al., 2000). Evidence obtained from studies culturing bacteria supports the view that the most common pathway of BIAC is the ascending route (Romero and Mazor, 1988; DiGiulio, 2012).

This study reviews and organizes systematically data published on the identity and frequency of detection of bacterial taxa found in the intra-amniotic space of women who delivered preterm. Its focus is on the dramatic advance of the knowledge of the bacterial communities present in the genital microbiota of pregnant women made in the last 18 years by non-cultivation, high-throughput techniques of analysis, and the potential contributions systematic investigations of the female genital microbiome can make to preventing PTB.

## Sources and study selection

### Database searches

An initial search of PubMed was conducted employing the broad concepts: “pregnancy,” “preterm birth,” and “intrauterine infection” or “chorioamnionitis,” as well as appropriate synonyms and truncations via the Boolean search method. The searches returned up to 1242 titles. Adding the term “bacteria” reduced the number of publications to 328. The titles and abstracts of this list were examined, and a selection was made following the inclusion criteria for studies that: (1) were published between 1995 and 2013; (2) contained data on bacterial taxa in the uterus of pregnant women delivering preterm; and (3) employed cultivation or molecular methods of identification of bacterial species. Excluded were publications: (a) in a language other than English; and (b) that did not specify the type of microbes involved in the invasion of the amniotic space.

Perusal of the selected papers and references therein yielded 13 papers containing information required for this review (Jalava et al., 1996; Markenson et al., 1997; Gardella et al., 2004; DiGiulio et al., 2008, 2010a, b,c; Han et al., 2009; Jones et al., 2009; Srinivasan et al., 2009; Zhou et al., 2010; Marconi et al., 2011). The data from the selected studies were extracted to construct a database of intra-uterine bacterial taxa or genera identified in PTB and the frequencies at which they were found.

### Phylogenetic organization

Phylogenetic trees of various bacterial phyla and their respective orders, families, genera and species, were employed as templates to classify the identity of intrauterine bacterial genera and species found in pregnant women who gave birth before term. The process served to arrange bacterial taxa into appropriate phyla and orders according to the NCBI taxonomy database.

### Analyses

The frequency at which a taxon or genus was present was determined from the data in the 13 publications included in this study by adding the number of women with intra-uterine infections who delivered preterm in which the taxon was found. The number of taxa in different phyla and orders were calculated in a similar way from the data in these publications.

## Results and conclusions

The review includes 761 women delivering before term of which 349 (46%) presented with an intra-uterine infection. The use of non-culture direct-detection techniques has increased by ~5-fold the number of taxa known to be present in intrauterine infections during pregnancy.

Meta-analyses of randomized trials evaluating antibiotic treatments report statistically significant prolongation of pregnancy associated with the use of antibiotics in women with preterm labor and intact membranes (King and Flenady, 2002), and reduction in the delivered number of babies within 48 h in preterm premature rupture of the membranes (Kenyon et al., 2010). Thus, there is a strong association between the presence of some bacteria in the intra-amniotic cavity and PTB.

**Figure 2:**
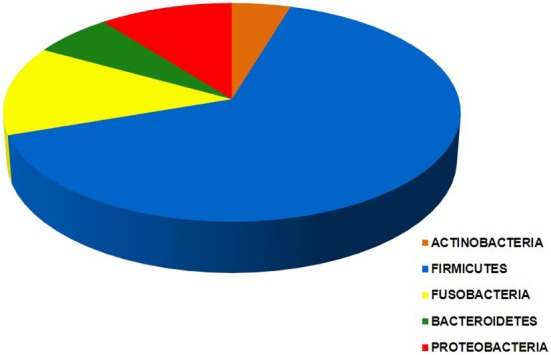
**Chart of the five phyla of the intra-uterine bacteria of 349 women with intra-amniotic infection who gave birth preterm.** Each segment is proportional to the number of women in which bacteria of a given phylum are found: Actinobacteria (25), Firmicutes (343), Fusobacteria (71), Bacteroidetes (20), and Proteobacteria (58). The data indicate that taxa from more than one phylum were present in most of these women.

Bacteria belonging to a total of 5 phyla and 16 orders were found in the intra-uterine microbiota of the 349 pregnant women with intra-amniotic infection (Table 1, Figure 2). They belonged to 44 genera and more than 87 different taxa (identification of some bacteria was performed only at the genus level) (Table 1). The highest frequencies were determined for genera of the order Mycoplasmatales (59%) and Lactobacillales (25%) (Table 1).

**Table 1:** **Bacteria found in the intra-uterine microbiota of the 349 pregnant women with intra-amniotic infection**.

**Phylum**	**Order**	**Species**	**Frequency**
			**(*n*)**
Actinobacteria	Actinomycetales	*Actinomyces* spp.	1
		*Brachybacterium* spp.	1
		*Corynebacterium ammoniagenes*	1
		*Corynebacterium amycolatum*	1
		*Corynebacterium tuberculostearicum*	1
		*Mobiluncus mulieris*	1
		*Propionibacterium acnes*	1
		*Propionibacterium* spp.	2
		*Rothia dentocariosa*	1
	Coriobacteriales	*Atopobium vaginae*	2
	Bifidobacteriales	*Bifidobacterium longum*	1
		*Bifidobacterium pseudolongum*	1
		*Gardnerella vaginalis*	11
Firmicutes	Clostridiales	*Clostridium hiranonis*	2
		*Clostridium perfringens*	2
		*Eubacterium halii*	1
		*Eubacteriun* spp.	1
		*Faecalibacterium* spp.	1
		*Filifactor alocis*	1
		*Finegoldia magna*	2
		*Peptoniphilus assacharolyticus*	4
		*Peptoniphilus lacrimalis*	1
		*Peptostreptococcus oralis*	1
		*Peptostreptococcus* spp.	14
		*Oribacterium sinus*	1
	Erypsipelotricales	*Coprobacillus* spp.	1
	Mycoplasmatales	*Mycoplasma hominis*	33
		*Ureaplasma parvum*	22
		*Ureaplasma urealyticum*	38
		*Ureaplasma* spp.	112
	Bacillales	*Listeria monocytogenes*	2
		*Staphylococcus aureus*	6
		*Staphylococcus epidermis*	1
		*Staphylococcus equorum*	2
		*Staphylococcus pettenkoferi*	2
		*Staphylococcus* spp.	6
	Lactobacillales	*Enterococcus faecalis*	1
		*Enterococcus spp.*	10
		*Lactobacillus crispatus*	3
		*Lactobacillus delbrueckii*	1
		*Lactobacillus gasseri*	1
		*Lactobacillus iners*	1
		*Lactobacillus* spp.	3
		*Streptococcus agalactiae*	37
		*Streptococcus anginosus*	11
		*Streptococcus mitis*	10
		*Streptococcus oralis*	4
		*Streptococcus pneumoniae*	3
		*Streptococcus salivarius*	1
		*Streptococcus* spp.	1
Fusobacteria	Fusobacteriales	*Fusobacterium gonidoformans*	1
		*Fusobacterium nucleatum*	31
		*Fusobacterium* spp.	4
		*Leptotrichia amnionii*	5
		*Leptotrichia* spp.	17
		*Sneathia sanguinegens*	13
Bacteroidetes	Bacteroidales	*Bacteroides fragilis*	4
		*Bacteroides xylanosolvens*	1
		*Bacteroides* spp.	3
		*Prevotella bivia*	4
		*Prevotella copri*	2
		*Prevotella oris*	1
		*Prevotella* spp.	3
	Flavobacteriales	*Bergeyella* spp.	1
		*Myroides* spp.	1
Proteobacteria	Campylobacterales	*Campylobacter ureolyticus*	3
		*Campylobacter* spp.	1
	Neisseriales	*Kingella denitrificans*	1
		*Neisseria cinerea*	1
		*Neisseria subflava*	1
		*Neisseria* spp.	1
	Burkholderiales	*Delftia acidovioans*	1
	Pasteurellales	*Haemophilus haemoglobinophilus*	1
		*Haemophilus influenza*	10
		*Haemophilus parainluenza*	2
		*Haemophilus quentini*	1
	Enterobacteriales	*Citrobacter koseri*	1
		*Enterobacter aerogenes*	1
		*Enterobacter* spp.	2
		*Escherichia coli*	25
		*Proteus mirabilis*	4
		*Shigella* spp.	2

The frequency is given as the number of women (n) carrying a particular species.

Bacteria of the phylum Firmicutes were the most abundant and were detected in 343 women with infection included in this study; the second most common phylum among these women was Fusobacteria, found in 71 subjects (Figure 2). The phyla with larger number of different orders and taxa were Firmicutes and Proteobacteria (Table 1).

Taxa of the order Mycoplasmatales were found in 205 (58.7%) women, and bacteria of the genus *Ureaplasma* were detected in 172 women (49%). Recognized genital pathogenic species were found at high frequencies, e.g., *Ureaplasma uralyticum* (11%), *Streptococcus agalactiae* (11%), *Mycoplasma hominis* (9%) and *Fusobacterium nucleatum* (9%) (Table 1). These results are in broad agreement with previous more limited knowledge of BIAC. A review of the pathogens involved in sepsis in neonatal intensive care units found that the majority were mixed genital tract flora (Garland and Bowman, 2003). Meta-analyses of antibiotic administration to women with bacterial vaginosis showed an association of the treatment with a significant reduction in the incidence of PTB and low weight babies (Smaill, 2001). This work indicated taxa present at higher frequencies belonged to bacteria normally found in the urogenital and gastrointestinal tracts; a result that supports the view that most cases of chorioamnionitis arise from pathogens ascending from the vagina. Thus, it is reasonable to hypothesize that preventing ascending genital tract infection and the initiation of inflammatory cascades will reduce PTB, neonatal fever and other morbidities.

Pathogens that are ordinarily found in the gastrointestinal tract and may reach the vagina, also can cause haematogenous invasion of the uterus. *Listeria monocytogenes* crosses the mucosal barrier of the intestine to disseminate haematogenously to any site, with a unique tendency to infect the fetoplacental unit (Baud and Greub, 2011). Generally, the bacteremia manifests clinically as non-specific influenza-like symptoms, and may remain asymptomatic. A review of 36 cases of maternofetal listeriosis showed that the mothers generally were affected mildly by the infection. Twelve pregnancies ended with abortion or stillbirth; among the children born alive, 15 were diagnosed with bacteremia/septicemia, 3 with pneumonia, 3 with neonatal meningitis, 1 died, and 3 were unaffected (Smith et al., 2009).

There is evidence to support the hypothesis that bacterial infections at sites distant from the urogenital tract, in particular the oronasal cavity and the respiratory tract, may be important causes of preterm labor probably through the activation of abnormal inflammatory responses within the uterus and intrauterine tissues. Data from clinical and animal studies on maternal periodontal status combined with a biologically plausible mechanism provide strong evidence for a negative impact of periodontal infection on pregnancy outcome (Baskaradoss et al., 2012).

### Biac by oronasal microflora

This review showed that a number of taxa found in periodontal disease were associated with PTB; they had a frequency of *ca*. 13% in women delivering before term. Identified taxa of the oronasal habitat included *Bergeyella* spp., *Dialister* spp., *Fusobacterium nucleatum*, *Oribacterium sinus*, *Peptostreptococcus oralis, Prevotella oris, Rothia dentocariosa, Streptococcus oralis*, *Streptococcus salivarius*, *Veillonella parvula*, and *Veillonella* spp. It should be noted that many of these taxa are frequently detected in faces, e.g., *S. salivarius* and *F. nucleatum*; and in the vagina, e.g., *P. oralis*, *P. oris*, *S. salivarius*, *Veillonella* spp., and *Dialister* spp.

Using 16S and 23S rDNA molecular methods, a *Bergeyella* spp. strain detected in the amniotic fluid of a pregnant woman with clinical intrauterine infection and histologic necrotizing acute and chronic chorioamnionitis was detected also in the subgingival plaque of the patient but not in her vaginal tract. The results suggested that the woman's intrauterine infection with this *Bergeyella* strain originated from the oral cavity (Han et al., 2006). *Capnocytophaga* spp. are part of the normal human oral bacterial flora, but as opportunistic pathogens can produce extra-oral infections including septicaemia and, less commonly, chorioamnionitis and neonatal infections. Evidence suggests that a number of cases of intra-amniotic infection with this bacterium occurred by the ascending route, but several cases that involved early-onset of sepsis due to *Capnocytophaga* spp. infection yielded no vaginal cultures of this bacterium suggesting haematogenous spread from the oral cavity (Lopez et al., 2010).

Evidence that the oral pathogen *F. nucleatum* may be transmitted haematogenously to the placenta and cause adverse pregnancy outcomes was obtained in pregnant mice injected intravenously with the bacterium. *F. nucleatum* colonizes the placenta and proliferates rapidly, inducing fetal death by localized infection inside the uterus; the bacterial infection was restricted inside the uterus, without spreading systemically (Han et al., 2004).

The bacterium *Rothia dentocariosa* is a common inhabitant of the human oral cavity where it rarely causes serious infections; it has been associated with endocarditis, pneumonia, septicemia, and abscesses in adults. *R. dentocariosa* caused septicemia in a neonatal infant with meconium aspiration syndrome (Shin et al., 2004), and was detected in the blood of a stillborn baby (Karlsson and Jacobsson, 2005). Its presence in the vagina is rare; only one woman with no signs of infection was reported in a study comprising 394 subjects (Ravel et al., 2011). The infrequent detection of this bacterium in the vagina makes it plausible that in the cases of neonatal septicemia and the stillborn infant, the access to the intra-amniotic cavity occurred via the haematogenous pathway.

*Streptococcus oralis* has been found in the intra-amniotic cavity (Jalava et al., 1996), and was associated with PTB in a study comparing women delivering preterm or at term (Skuldbø et al., 2006), but the routes of invasion were not established in these studies.

A clinical study of 812 deliveries from a cohort study of pregnant mothers entitled “Oral Conditions and Pregnancy” demonstrated that both antepartum maternal periodontal disease and incidence/progression of periodontal disease are associated with PTB and growth restriction after adjusting for traditional obstetric risk factors. The results support the concept that maternal periodontal infection in the absence of a protective maternal antibody response is associated with systemic dissemination of oral organisms that translocate to the fetus resulting in prematurity (Madianos et al., 2001). Analysis of oral bacteria in the amniotic cavity of women delivering preterm agreed with the results of a meta-analysis of 12,047 pregnant women that found a 2.73 overall odds ratio (95% CI: 2.06–3.6, *p* < 0.0001) of giving premature birth to a child for mothers with periodontitis (Konopka and Paradowska-Stolarz, 2012). These findings provide support for the hypothesis that haematogenous dissemination of oronasal bacteria is probably one of their routes of access to the amniotic cavity.

### Biac by respiratory tract microflora

Haematogenous spreading of infections from the upper or lower airways to the placenta may occur at any stage of the pregnancy (Sandu et al., 2013). The data collected in this review indicated that in the intra-amniotic cavity of women giving birth preterm were found at low frequencies bacteria that colonies the respiratory tract such as *Haemophilus influenza*, *Haemophilus parainfluenza*, and *Streptococcus pneumoniae*.

Although these and other respiratory tract pathogens have been found in the vaginal microbiota (Ravel et al., 2011), in mothers with acute respiratory infection induced by highly virulent pathogens, the infection may spread haematogenously to the placenta inducing spontaneous and/or septic abortions, premature births, fetal damage or intra-uterine fetal death (Sandu et al., 2013). Pregnant women with pulmonary tuberculosis have higher odds of PTB (Asuquo et al., 2012). In a stillbirth where the mother had an upper respiratory infection of *F. nucleatum*, the bacterium was isolated from the placenta and the infant, and the same clone was identified in her subgingival plaque, but not in the vagina or rectum (Han et al., 2010).

*Haemophilus influenzae* is primarily responsible for neonatal meningitis and respiratory tract infections in children. It has a low prevalence rate in genital tract cultures and rarely causes acute endometritis, but intra-amniotic infection and positive blood cultures have been reported (Shute and Kimber, 1994). A sepsis secondary to an acute *H. influenzae* infection led to placental abruption and spontaneous abortion (Calner et al., 2012); also a case has been reported with the uterus as the primary focus of sepsis with presence of the bacterium in blood but not in the vagina (Martin et al., 2013). Vertical transmission of *H. influenzae* appears to be the most common route of infection of the fetus, but ascending infections are less common in *H. parainfluenzae* infections (Garcia et al., 1997).

Reports indicate involvement of oral and, less commonly, respiratory tract pathogens in intra-uterine infections. The presence of the same bacterium at the original point of infection and in the uterus supports a causal relationship and a role for haematogenous BIAC during pregnancy that could lead to PTB.

*Streptococcus pneumoniae* is a common pathogen of the general population; it is a frequent cause of pneumonias, meningitis, bacteremia, and sepsis. This bacterium is uncommon in the vaginal flora and is rarely associated with gynecologic infections, but has been found in intra-amniotic infections causing septic abortions with no evidence of vaginal infection (Liang and Yeh, 2005). In a study of 29 cases of *S. pneumonia* infection of neonates, one mother had bacterial infection at delivery and clinical amnionitis (Hoffman et al., 2003).

The vast majority of the genera identified in intra-amniotic infections belong to bacteria found in the indigenous human microbiota. Leaving out exogenously acquired bacteria, and considering that more than a trillion microbes inhabit body surfaces and cavities, and outnumber human cells by at least a factor of 10, the human body is a rich potential source for opportunistic BIAC. Pathogens residing in body sites that could access the ascending migration or haematogenous route will influence the diversity and abundance of bacteria in the amniotic fluid.

## Future research

Amongst newborns, low and very low weight infants are at the highest risk of early death or disability, thus, a major focus of research in Obstetrics should be a better understanding of the processes that lead to PTB and the development of preventive interventions (Lockwood, 2013).

“Future efforts to reduce the rate of PTB depend upon gaining an improved understanding of the causative mechanism(s), determining differences in individual susceptibility, and identifying specific early-stage biomarkers that will allow the development of novel and timely intervention strategies.” (Hussein et al., 2011).

In the last 15 years, the significant progress made in the knowledge of the diversity of bacterial communities in the female genital tract and the role of bacterial infections in PTB wrought by novel sequencing techniques and bioinformatics tools, have made reduction of PTB a goal achievable by research directed toward prevention of BIAC by pathogens.

Considering the limitations of studies based on bacterial cultivation to reveal all the microflora present, new comprehensive investigations employing non-culture methods and state-of-the-art sequencing analyses are required to establish the intra-uterine microbiome in health and disease. A complete census of the intra-uterine microbiota during pregnancy conducted concurrently with a census of the vaginal microbiome will serve to outline the characteristics of the bacterial communities in the female genital tract; in particular, the elucidation of the microbial intra-uterine populations in healthy pregnant women, as well as the contribution of ascending infections to BIAC.

Future investigations that establish with more accuracy the bacterial taxa found in association with PTB, as well as their routes of invasion of the intra-amniotic cavity will provide important knowledge to support the development of earlier and more specific diagnostic methods of maternal genital infections. This will result in better targeted and more effective treatments, including many infections that presently are clinically silent and can cause significant morbidity in fetuses and infants. A full understanding of the female urogenital microbiome will render these infections amenable to intervention and will have an impact in the prevention of PTB.

### Conflict of interest statement

The authors declare that the research was conducted in the absence of any commercial or financial relationships that could be construed as a potential conflict of interest.
